# Tonic and Phasic Smooth Muscle Contraction Is Not Regulated by the PKCα - CPI-17 Pathway in Swine Stomach Antrum and Fundus

**DOI:** 10.1371/journal.pone.0074608

**Published:** 2013-09-18

**Authors:** Yu Zhang, Meghan E. Hermanson, Thomas J. Eddinger

**Affiliations:** 1 Department of Biological Sciences, Marquette University, Milwaukee, Wisconsin, United States of America; 2 Department of Biology, Bradley University, Peoria, Illinois, United States of America; Temple University School of Medicine, United States of America

## Abstract

Regulation of myosin light chain phosphatase (MLCP) via protein kinase C (PKC) and the 17 kDa PKC-potentiated inhibitor of myosin light chain phosphatase (CPI-17) has been reported as a Ca^2+^ sensitization signaling pathway in smooth muscle (SM), and thus may be involved in tonic vs. phasic contractions. This study examined the protein expression and spatial-temporal distribution of PKCα and CPI-17 in intact SM tissues. KCl or carbachol (CCh) stimulation of tonic stomach fundus SM generates a sustained contraction while the phasic stomach antrum generates a transient contraction. In addition, the tonic fundus generates greater relative force than phasic antrum with 1 µM phorbol 12, 13-dibutyrate (PDBu) stimulation which is reported to activate the PKCα – CPI-17 pathway. Western blot analyses demonstrated that this contractile difference was not caused by a difference in the protein expression of PKCα or CPI-17 between these two tissues. Immunohistochemical results show that the distribution of PKCα in the longitudinal and circular layers of the fundus and antrum do not differ, being predominantly localized near the SM cell plasma membrane. Stimulation of either tissue with 1 µM PDBu or 1 µM CCh does not alter this peripheral PKCα distribution. There are no differences between these two tissues for the CPI-17 distribution, but unlike the PKCα distribution, CPI-17 appears to be diffusely distributed throughout the cytoplasm under relaxed tissue conditions but shifts to a primarily peripheral distribution at the plasma membrane with stimulation of the tissues with 1 µM PDBu or 1 µM CCh. Results from double labeling show that neither PKCα nor CPI-17 co-localize at the adherens junction (vinculin/talin) at the membrane but they do co-localize with each other and with caveoli (caveolin) at the membrane. This lack of difference suggests that the PKCα - CPI-17 pathway is not responsible for the tonic vs. phasic contractions observed in stomach fundus and antrum.

## Introduction

The mechanism(s) determining tonic (sustained) vs. phasic (transient) smooth muscle (SM) contractions remains unresolved. Diverse innervation and circulating signaling molecules in addition to unique receptor and protein (isoform) expression and intracellular signaling pathways complicate the issue. Not unexpectedly, there are multiple proposed hypotheses to explain tonic force maintenance in SM including: slowly- or non-cycling smooth muscle myosin cross-bridges called latch bridges [1], [2], [3], [4]; recruitment of non-muscle myosin II [5], [6], [7]; formation of caldesmon or calponin dependent actin-to-myosin cross-links [8], [9]; formation of cytoskeletal force-bearing structures [10], [11], [12]; and regulation of myosin LC_20_ phosphorylation via second messenger pathways affecting myosin light chain kinase (MLCK) and myosin light chain phosphatase (MLCP) activity [13], [14], [15], [16], [17], [18]. This last category is of interest because of the reported differential expression, localization and regulation of these second messenger proteins in various SM tissues. Variable regulation of MLCK and MLCP activity could provide not only a means for Ca^2+^ sensitization, but also a possible mechanism for tonic vs. phasic contraction. Inactivation of MLCP via the PKCα –CPI-17 pathway would allow for maintained high myosin light chain (MLC_20_) phosphorylation and thus a sustained contractile force. A failure to inactivate MLCP would reduce MLC_20_ phosphorylation and force, resulting in a transient contraction. Stomach fundus SM generates a tonic (sustained) contraction while stomach antrum SM generates a phasic (transient) contraction in response to a variety of stimuli.

Ca^2+^ sensitization, which involves the activation of G-protein-coupled second messenger pathways that sensitize the contractile proteins to [Ca^2+^_i_] by inhibiting MLCP, can occur by any of numerous pathways. One of the reported pathways for inhibition of MLCP activity includes the protein kinase C/C-kinase activated PP1 inhibitor protein of 17 kDa (PKC/CPI-17) pathway [19], [20]. G-coupled protein receptor (GCPR) activation resulting in activation of phospholipase C (PLC), hydrolyzes phosphatidylinositol 4,5-bisphosphate (PIP_2_) to produce inositol 1,4,5-trisphosphate (IP_3_) and diacylglycerol (DAG). DAG is known to activate PKC which can cause phosphorylation of CPI-17 to selectively inhibit MLCP (reviewed for visceral SM in [17]). PKC is believed to be regulated by its activation and spatial distribution within the cell, the latter being controlled by anchoring proteins that bind to and restrict the location of PKC to specific regions of the cell [21], [22]. For example, reports of constitutively active L-type Ca^2+^ channels and their regulation by PKC [23], [24] suggests that PKC is localized near the membrane in smooth muscle cells. The cellular function of CPI-17 in the cell could vary depending on its level of protein expression and whether it is located near the plasma membrane where PKCα is located/activated, or associated with the contractile myosin thick filaments where MLCP would be located to de-phosphorylate myosin light chain 20 (MLC_20_).

The expression of PKCα and CPI-17 and their spatial-temporal re-distribution upon tissue activation have been proposed in Ca^2+^ sensitization of smooth muscle tissues (for review [14], [15], [16], [17]). Thus it is possible that differences in their expression and cellular localization/translocation could be involved in determining tonic vs. phasic contractile responses. For these two proteins to be part of a pathway involved in inhibiting MLCP activity during tissue activation, and thus causing a tonic or phasic response, they need to be expressed at appropriate levels and positioned at the correct location in the cell at the correct time. Because PKC is activated by DAG (a membrane bound phospholipid), it must also be at the membrane at least temporally for this to happen. And if CPI-17 is going to inhibit the MLCP from dephosphorylating MLC_20_ on the thick filaments, CPI-17 at some point needs to be in the cytosol associated with the thick filaments present there. With these two steps occurring in two spatially distinct regions of the cell, one or both of these proteins has to translocate to the other region for them to interact and complete the pathway.

The purpose of this study was to test the hypothesis that the PKCα - CPI-17 Ca^2+^ sensitization pathway is involved in determining tonic (sustained) and phasic (transient) contractile responses in smooth muscles. To do this we determined the protein expression and spatial-temporal distribution of PKCα and CPI-17 in the phasic stomach antrum and tonic stomach fundus under relaxed and activated conditions. The results showing no significant difference in expression of either of these two proteins, nor in their peripheral distribution between these two tissues are inconsistent with the hypothesis that the PKCα- CPI-17 Ca^2+^ sensitization pathway is the determining factor for the tonic vs. phasic contractile responses observed in these tissues.

## Methods

### Organ and Tissue Handling

Swine tissues (stomachs) were obtained from Hansen Meat Service (Franksville, WI) and put in cold physiological salt solution (PSS; (in mM): 140.1 NaCl, 4.7 KCl, 1.2 Na_2_HPO_4_, 2.0 MOPS (pH 7.4), 0.02 Na_2_EDTA, 1.2 MgSO_4_, 1.6 CaCl_2_, and 5.6 glucose). Stomachs were cleaned of blood, loose connective tissue, and in some cases, the mucosa, and frozen immediately or stored in PSS in the refrigerator for 0–2 days. Some organs were fresh frozen as soon as possible following post-mortem (60–90 minutes). Some organs were incubated in PSS and/or stimulated with 1.0 µM CCh or PDBu (Sigma) at 37°C for different time points prior to freezing. Variable incubation times and agonist concentrations were also tested. For immunohistochemistry all tissue was stored frozen until sectioned and immunoreacted. Freezing in all cases involved placing pieces of tissues or tissue strips in isopentane cooled in liquid nitrogen followed by storage at −80°C. Five to six µm sections of the frozen tissues were cut on a Leica CM1900 cryostat, picked up on glass slides and stored frozen (0–1 days) until immunoreacted.

### Immunoreactions and Reagents

The antibodies used were obtained from the following sources: PKCα (H-7 and C-20) and CPI-17(H-60) from Santa Cruz Biotech, Santa Cruz CA; Vinculin and Talin from Sigma, Saint Louis, Missouri; Caveolin 1 from BD Biosciences, San Jose, CA; Cy2 and Cy3 Donkey anti mouse or rabbit secondary’s from Jackson ImmunoResearch, West Grove, PA; Alexa Fluor 594-phalloidin and DAPI from Molecular Probes, Eugene, OR. All of these antibodies have been used previously in our laboratory for immunohistochemistry and western blotting where they show specificity for a band of the correct molecular weight for the respective protein indicated [25], [26], [27]. Negative controls where the primary antibody is not included show no reactivity for these bands and no immunofluorescence on tissue sections.

Frozen tissue sections picked up on glass slides were thawed at room temperature and then fixed with 2% paraformaldehyde for 10 minutes, permeabilized in 0.5% Triton X-100 for 10 minutes and blocked with 5 mg/ml BSA for 1 hour prior to reacting with the primary antibody overnight (4°C) and then the appropriate secondary antibody for one hour at room temperature. After the secondary antibody, the tissues were incubated with DAPI (0.5 µM), phalloidin (10–50 nM) or DAPI/phalloidin as appropriate for staining nuclei and/or filamentous actin. Multiple washes were used following the primary and secondary incubations, and the counterstaining. Cover glasses were mounted using buffered 75% glycerol with 0.2% n-propyl gallate to minimize fading. All immunoreacting solutions were made in PBS-Tween [(in g/liter: NaCl 8.0, KH_2_PO_4_ 0.2, Na_2_HPO_4_ 1.15, KCl 0.2,), 1% tween-20, pH 7.4] with 0.1% BSA. Negative controls included 0.1%BSA without the primary antibody, and showed no immunoreaction.

### Microscopy

Sections were observed using an Olympus IX70 microscope with epifluorescence illumination. Digital images were taken with a 16 bit Princeton Instruments (Princeton, NJ) CCD camera, controlled through a PCI board via IPLab for Windows on a PC (Ver. 3.6, Scanalytics; Fairfax, VA). Images were taken using either a 100× (1.3 NA) or 60× (1.25 NA) oil lens or a 40× (0.9 NA) air lens and stored on the PC. Emission filters used were 405, 490 and 570 nm. Sections were also viewed using a Nikon confocal microscope (Nikon A1 confocal Eclipse Ti). The objectives used were 100× (1.4 NA) oil lens at 425, 488 and 561 nm. Similar results were observed in immunofluorescence distributions using these two different systems.

### Image Analysis of Distribution of PKC/CPI-17 of Individual Cells in Tissues

Profiles of fluorescence intensity were taken for individual cells in transverse tissue sections. Sections were observed at low magnification (10–20x) to avoid areas of apparent artifacts (tissue folding, freeze damage, etc). In an artifact free area the magnification was increased to 40–100x, and pictures were taken at these higher magnifications. Three different areas within one tissue section were chosen to take pictures. Z-stack series were taken individually for each of the three color channels used. 1 µm thick Z sections were taken, and 15–25 Z sections were taken for each tissue section (figures S1 & S2 show Z-stack examples of CPI-17 immunoreactivity in antrum with and without tissue activation). Each Z stack series was examined for each section to identify the center z image, and this image was converted to a.bmp file, which was imported to NIS-Elements AR 3.0 (Nikon) on a PC to analyze the data.

Profiles of PKCα and Phalloidin fluorescence intensity were obtained for individual cells in transverse tissue sections. A line was drawn across the center of the image field. Ten cells crossed by the line were selected for measurements. Based on our previous protocols [25], the first five pixels (∼0.7 µm) on either side of the cell where the phalloidin intensity increased sharply from baseline were defined as the periphery of the cell. For cytosolic measurements, a line was placed at least 2 µm away from the cell periphery. Intensity measurements were made using a region of interest (ROI) roughly at the center of the cell (for cytosolic measurements), or along the cell’s membrane (for peripheral measurements). For consistency, the size of the ROI is kept constant for the peripheral and cytosolic measurements. Cells in the section with very small diameters (not able to measure cytosolic ROI at least 2 µm from the periphery) or with nuclei present (visible DAPI staining where spatial limitations and perinuclear organelles could confound the distribution) were excluded from analysis. For PKCα, the ratio of the average intensity of PKCα at the periphery over the total PKCα intensity (sum of PKCα intensity from ROI at periphery and ROI in cytosol) was used. Ten cells per field and three different fields were subjected to measurement for data analysis for each tissue region, i.e. thirty cells were counted and averaged for each sample (n = 1). The final sample size was n = 5.The same procedures were applied to measure the intensity of CPI-17, except that only one field of 10 cells were used for each sample (n = 1), with a final sample size of n = 3.

### Mechanical Measurements

Immediately before use, tissue strips were cut and clamped on each end, with the clamps secured between hooks on a stationary metal rod and an isometric force transducer (Harvard Apparatus, Holliston, MA), in PSS bubbled with 95% O_2_/5% CO_2_ in water-jacketed muscle chambers (Radnoti Glass Technology, Monrovia, CA) at 37°C. The length of each strip was varied by repositioning the stationary metal rod.

Smooth muscle tissue strips (stomach antrum and fundus) were equilibrated for 1 h and stretched to a passive tension approximating Lo using an abbreviated length-tension curve. To contract tissues, PSS was replaced with K^+^-PSS (109 mM KCl is substituted iso-osmotically for NaCl) [28]. The muscle strips were activated repeatedly with stretching of the tissue between each activation, until peak force no longer showed a significant increase over the previous contraction. Chambers were flushed three times with PSS following each tissue activation. At least two repeatable successive K^+^-PSS contractions were used to get a standard force trace with a 10 minute rest between each contraction before starting the experiment. The tissues were then activated with 1 µM carbachol and relaxed again, as was performed during K^+^-PSS contractions. Subsequent contractions all included 1 µM phentolamine and propranolol to block α- and β - adrenergic receptors, respectively. Following the final 1 µM CCh contraction and wash, the tissues were activated with 1 µM PDBu to record their mechanical response.

### Analysis of Force Data

Voltage signals from force transducers were digitized by PowerLab 400 or 4 SP hardware (ADInstruments, Castle Hill, Australia) visualized on a computer screen (Chart v3.6 or 4.0, ADInstruments) as force (g) at 10 Hz and stored by software command to a hard disk for later analyses. Figures were made with the spreadsheet program Excel 2000 (Microsoft, Redmond, WA).

### Gel Electrophoresis and Western Blotting

Protein expression was analyzed as described previously [29]. Tissues were homogenized at 50 mg/ml in 0.125 M Tris, 2% sodium dodecylsulfate (wt/vol), 20% glycerol, 0.1% bromophenol blue (wt/vol) and 20 mM dithiothreitol. Proteins were resolved on low cross-linking sodium dodecylsulfate gels [30] and immunoblotting was performed as previously described [31]. The PKCα antibody recognized either a single band or more frequently a doublet at ∼76 kD while the CPI-17 antibody recognized a single band at ∼17 kD. These sizes are consistent with the expected size for these proteins based on gel mobility. To ensure accuracy of the western blots, loading curves were done for both stomach fundus and antrum samples over a 5-fold range of loadings (4–20 µl) for actin (coomassie blue staining) and PKCα and CPI-17 (western blotting). The results showed a linear relationship over this range for all three proteins from both tissues (R^2^>0.98 for all results, n = 3). Quantitation of PKCα and CPI-17 protein expression was done on western blots using 5–10 µl loadings that was within this linear range (n = 6).

### Statistics

**Figure 1:**
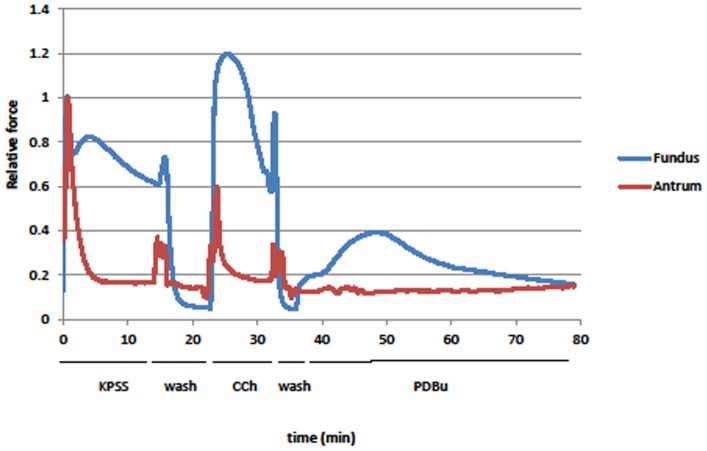
Force (normalized to peak force from KPSS stimulation) generated by stimulating with KPSS, 1 µM CCh, or 1 µM PDBu in antrum (red) and fundus (blue). Antrum contractile response is not maintained during the stimulation while that in the fundus is. PDBu generated a slow contraction in the fundus that is ∼40% of its peak KPSS force, but caused little to no contraction in the antrum. Deflections in the traces at the start of each wash are from changing the solution in the chambers. These are representative traces from 1 of 6 different animals used for mechanical measurements.

Statistical comparisons were carried out using MINITAB (Minitab Inc. State College, PA). A one sample t-test was used to test the distribution of PKCα/CPI-17 (a peripheral to total ROI content of 0.5 indicates a “uniform” distribution in smooth muscle cells (SMCs)) in antrum and fundus under resting condition. One way ANOVA was performed to test for PKC/CPI-17 distribution differences for the two tissues with different stimulating parameters. Two sample t-tests were used to test the significance of difference for expression level of PKCα or CPI-17 in antrum and fundus. A two-tailed F-test for equality of two standard deviations (SDs) determined a significantly lower SD in the ratio of caveolin to PKCα when compared to the SD of the ratio of vinculin to PKCα. A value of P<0.05 was considered significant for all statistical tests. No statistical tests were done on the force data and figure 1 is representative of similar data from 6 different animals. Western blot data is from six animals. PKCα distribution data is an average of thirty cells measured per animal and five animals were tested. CPI-17 distribution data is an average of 10 cells measured per animal and three animals were tested. Protein co-localization data is based on a ‘n’ of at least 20 cells from at least 3 animals.

## Results

Swine stomach fundus is a primarily tonic smooth muscle tissue that responds to stimulation with a sustained contraction while the antrum is a primarily phasic smooth muscle tissue that responds to stimulation with a transient contraction (Fig. 1). In tissues these responses are likely a result of direct stimulation of the smooth muscle as addition of 1 µM propranolol (β agonist blocker) and phentolamine (α agonist blocker) was used to prevent activation of adrenergic receptors due to potential release of sympathetic neurotransmitters. Stimulation of smooth muscle with PDBu is used routinely to cause smooth muscle contraction via PKC activation [32], [33], [34]. One µM PDBU causes a small slow contraction in stomach fundus strips (∼40% of K^+^ stimulation response) while the antrum shows essentially no response (<5% K^+^ stimulation) (Fig. 1). The difference between the sustained and transient responses of these two tissues to KPSS and CCh, and the presence or absence of a contractile response to PDBu stimulation could be due to the expression and/or spatial-temporal distribution of the downstream second messengers (PKCα and CPI-17) that are purported to be responsible for MLCP inhibition with PDBu stimulation. To examine this we measured the expression levels and determined the spatial-temporal distribution of PKCα and CPI-17 in these tissues.

**Figure 2:**
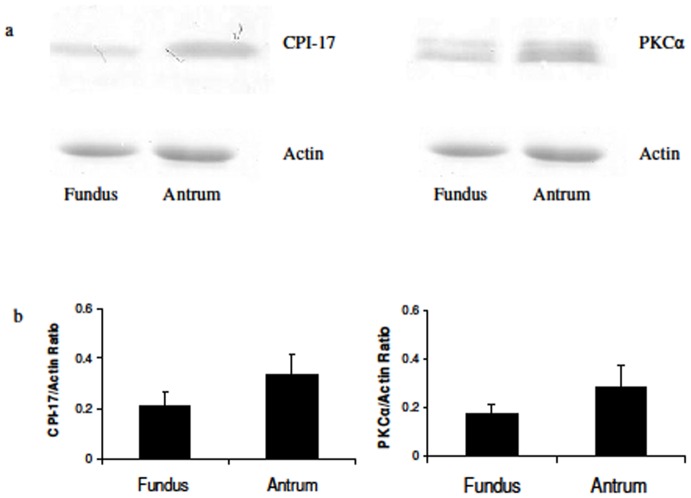
Protein expression of CPI-17 and PKCα in fundus and antrum. A: western blot results of CPI-17 (left) and PKC (right) expression in fundus and antrum with actin (coomassie blue stain) expression for each sample shown below. B: Quantitative data of gel/western blot results. The expression level of CPI-17 (left) and PKCα (right) were not significantly different (p>0.23 & p>0.28 respectively, n = 6) between the fundus and antrum.

Figure 2 shows western blot results for the expression of CPI-17 and PKCα in the stomach antrum and fundus. Tissues were processed controlling for protein concentration, and the results were calculated based on the intensity of the respective protein signal, and normalized to actin protein expression (Fig. 2). Both methods (only normalized data shown) indicated that neither the expression of CPI-17 protein nor that of PKCα protein is significantly different (p>0.23 & p>0.28 respectively, n = 6) when comparing these two SM tissues. Controls were done using a range of loadings to confirm the range of linearity of loading, and that samples used for quantitation were within this range (data not shown, see methods). Because there are no differences in the expression for these two proteins between these two tissues, we next proceeded to determine if differences in their spatial-temporal distribution could explain the difference in their responses to PDBu stimulation.

**Figure 3:**
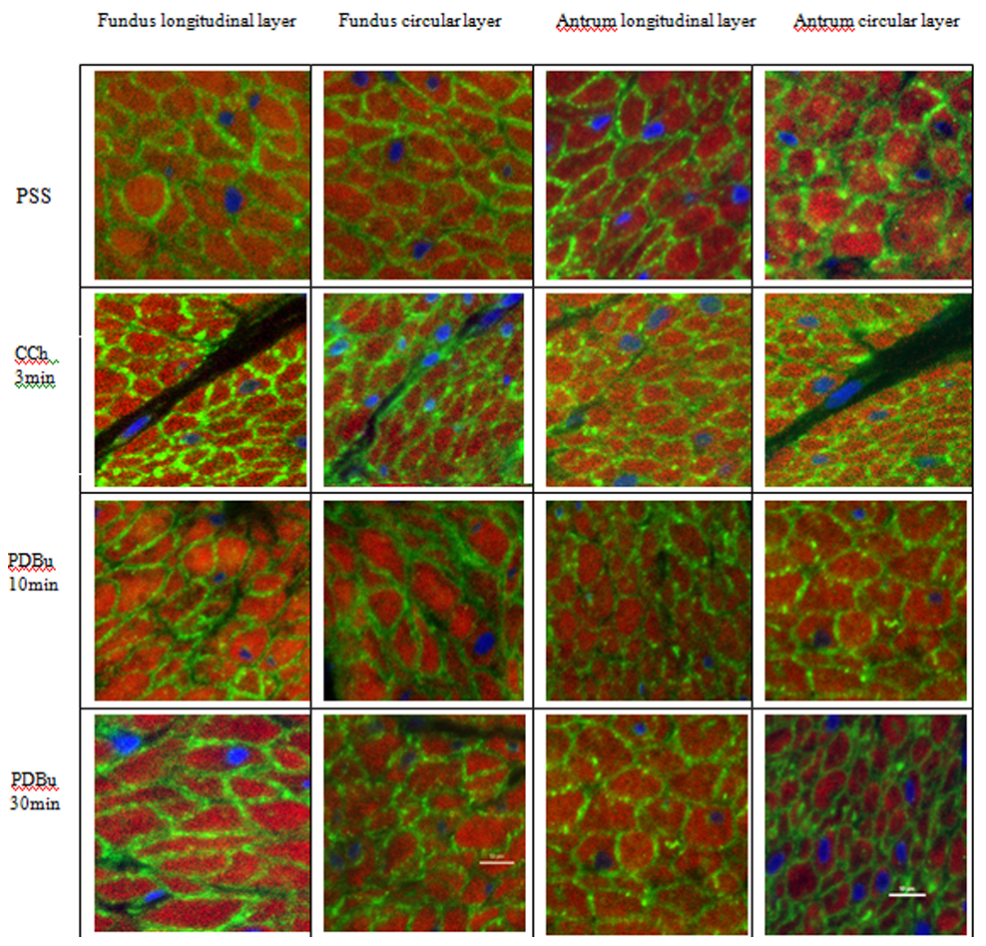
Confocal images of PKCα distribution in transverse sections of the longitudinal and circular layer of pig stomach fundus (left) and antrum (right) under relaxed (PSS) or stimulated (3′ CCh or 10 & 30′ PDBu) treatments. Tissues were immunoreacted for PKCα (green) and counterstained for filamentous actin (phalloidin - red) and nuclei (DAPI - blue). In all conditions, PKCα is located predominantly at the cell periphery near the plasma membrane. Scale bar −10 µm.

**Figure 4:**
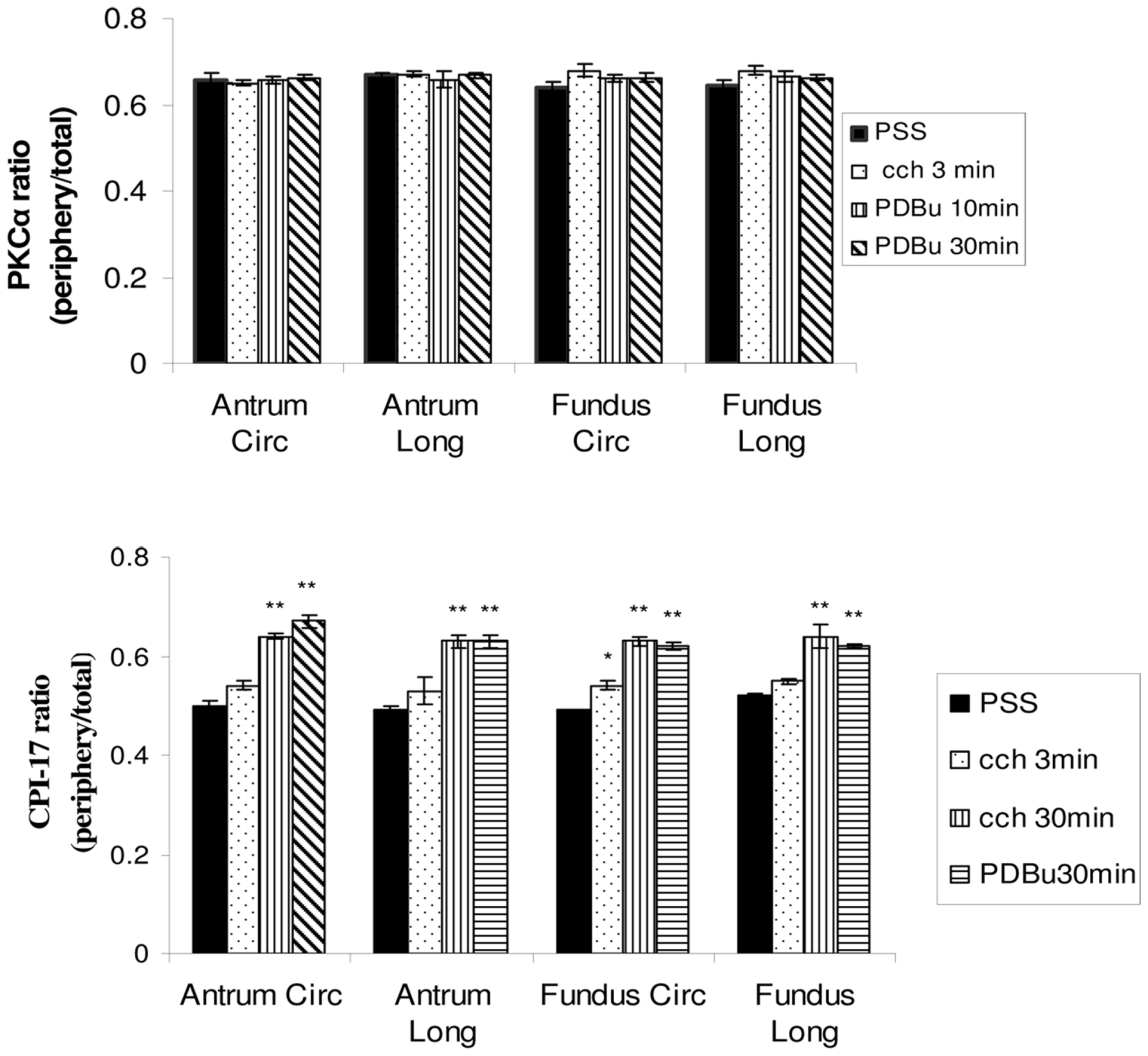
Quantitative results of the ratio of the PKCα (top panel) and CPI-17 (bottom panel) at the cells periphery to the total protein (peripheral+cytosolic) in the circular and longitudinal layers of the antrum and fundus. The ratio of PKCα in the relaxed condition (PSS) in both layers of antrum and fundus is significantly greater than 0.5, indicating a preferential distribution of PKCα near the plasma membrane in the relaxed condition. This ratio does not change significantly with different stimulation treatments, suggesting that PKCα maintains a primarily peripheral distribution in the cell at all times. (n = 5). The CPI-17 ratio in the relaxed condition (PSS) in both layers of antrum and fundus is not significantly different than 0.5, indicating a uniform distribution of CPI-17 throughout the cells. Three minutes of CCh stimulation does not change the ratio of CPI-17 except in the fundus circular layer. Both CCh (30′) and PDBu (30′) treatments cause a significantly redistribution of CPI-17 to the cell periphery near the plasma membrane (ratios are significantly greater than 0.5). * p<0.05, **p<0.01 (n = 3).

Figure 3 show immuohistochemical results for the distribution of PKCα in the longitudinal and circular layers of the fundus and antrum. Under resting conditions in relaxing solution when there is no force generation by the tissue, the PKCα appears to be uniquely distributed preferentially near the periphery of the SMCs (Fig. 3– green color, PSS). Stimulation of the tissues with 1 µM CCh (3′) or 1 µM PDBu (10 and 30′) does not alter this peripheral distribution of PKCα. The ratio of the distribution of the PKCα near the plasma membrane relative to total cellular PKCα was used to quantify possible changes in the distribution of this protein under these different conditions. Figure 4 shows the results as the ratio of the PKCα at the cell periphery relative to the total PKCα present in the cell (peripheral/(peripheral+cytosol), see methods). A ratio of 0.5 would indicate a “uniform” distribution of the protein throughout the cell. The PKCα ratio (peripheral/total) ranged from 0.64–0.68 in all the conditions examined. These values are significantly greater than 0.5 (p<0.05), indicating that PKCα is located primarily at the cell periphery (it is not “uniformly” distributed in the cell) and that this distribution does not change between the relaxed or stimulated conditions.

**Figure 5:**
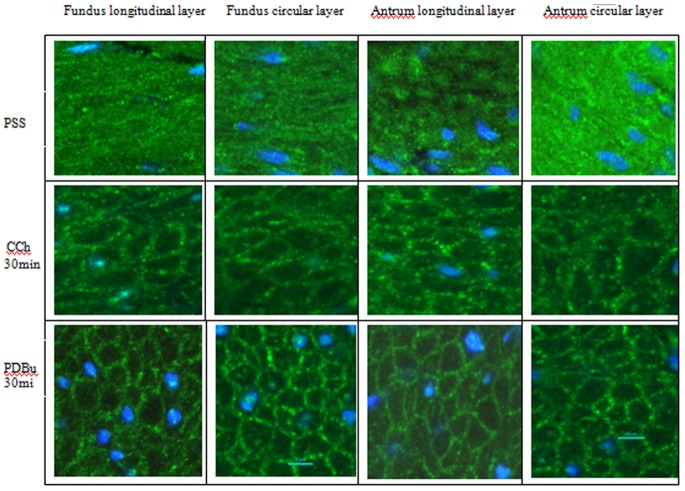
Confocal images of CPI-17 distribution in transverse sections of the longitudinal and circular layer of pig stomach fundus (left) and antrum (right) under relaxed conditions (PSS) or stimulated (30′ CCh or PDBu) treatments. Tissues were immunoreacted for CPI-17 (green) and nuclei (DAPI - blue). In relaxed condition (PSS), CPI-17 appears diffusely distributed throughout the cell. With either CCh or PDBu stimulation, CPI-17 appears predominantly located at the periphery near the plasma membrane. Scale bar –10 µm.

Because PKCα is proposed to activate CPI-17, we proceeded to determine the spatial-temporal distribution of CPI-17 in these tissues under similar conditions. Figure 5 show immuohistochemical results for the distribution of CPI-17 in the longitudinal and circular layers of the fundus and antrum. Under resting conditions in relaxing solution when there is no force generation by the tissue, the CPI-17 appears to be diffusely distributed throughout the SMCs (Fig. 5– PSS, fig. S1). Stimulation of the tissues with 1 µM CCh (30′) (but not 1 µM CCh for 3′, data not shown) or 1 µM PDBu (30′) results in a significant change in this distribution such that the CPI-17 now appears to be primarily at the periphery of the cell in a distribution similar to that observed for PKCα (Fig. 5, fig. S2). The ratio of the distribution of the CPI-17 near the plasma membrane relative to total CPI-17 (peripheral+cytosolic) was used to quantify possible changes in the distribution of this protein under these different conditions. Figure 4 shows the results as the ratio of peripheral-to-total CPI-17. The CPI-17 ratio (peripheral/total) ranged from 0.49–0.67 in all tissues and conditions examined. The ratio of CPI-17 peripheral/total in relaxed conditions is not significantly different than 0.5 (means 0.49–0.5; P>0.19) indicating that the CPI-17 is diffusely distributed throughout the smooth muscle cells under this condition. This does not change following 3′ of 1 µM CCh stimulation as there is still no significant difference from the relaxed conditions (means = 0.53–0.55; P>0.05) with the exception of the fundus tissues where the CPI-17 peripheral/total ratio is significantly greater (p<0.05) at 3′ (Fig. 4). With 30′ of either 1 µM CCh or 1 µM PDBu stimulation, the CPI-17 distribution becomes significantly greater than 0.5 for all tissues and layers (means = 0.62–0.67, P<0.01), indicating that CPI-17 is now located primarily at the cells periphery (it is no longer “uniformly” distributed throughout the cell), similar to the distribution of PKCα (Fig. 4).

**Figure 6:**
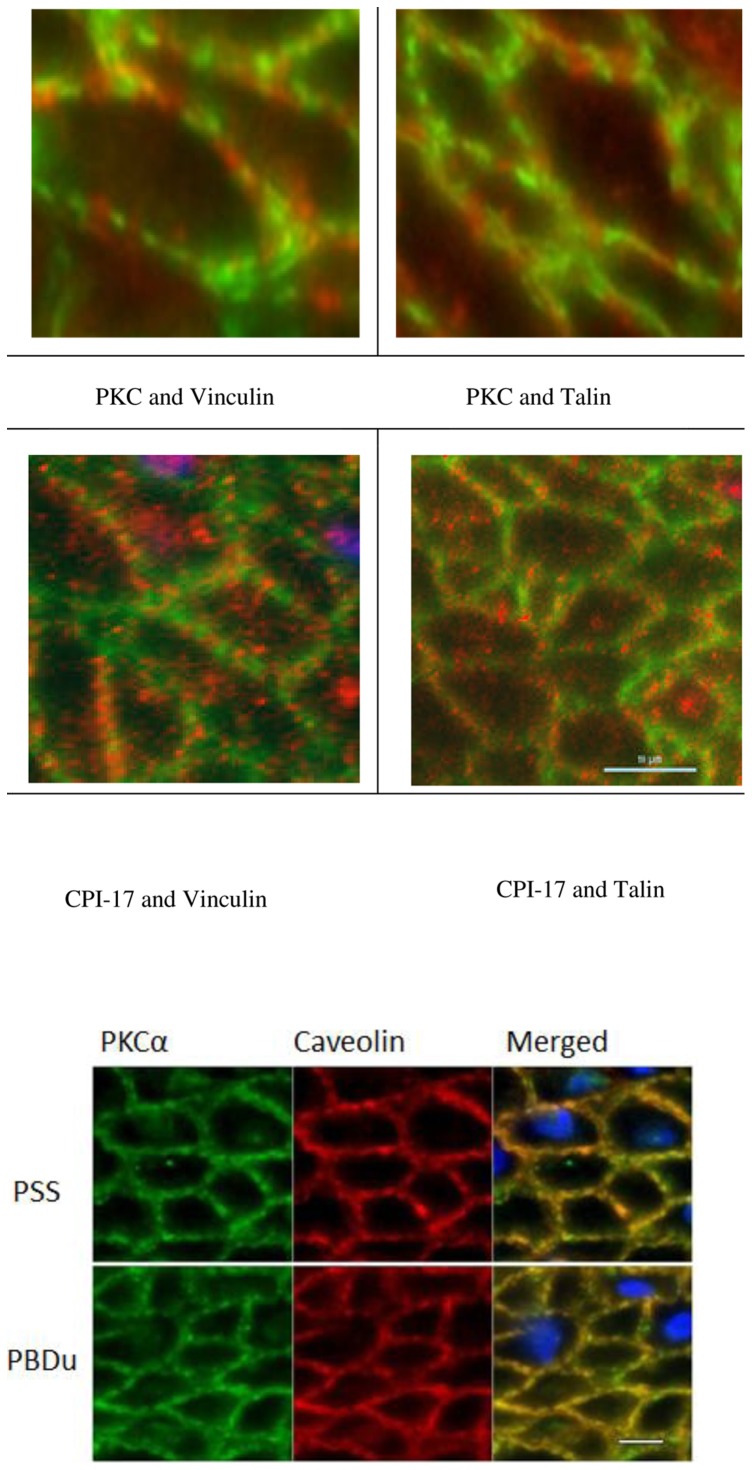
Confocal images of pig fundus (upper panel) in relaxed condition (top row) and with PDBu (30′) stimulation (lower row). Double labeling for PKCα (green) with vinculin (red, upper left) or talin (red, upper right) or CPI-17 (green) with vinculin (red, lower left) or talin (red, lower right). Vinculin and talin are adherens junction associated proteins that appear to have an alternating distribution with PKC and CPI-17, suggesting that these proteins are not localized to the same domains near the plasma membrane. Note that not all of the PKCα or CPI-17 is located near the plasma membrane. Lower panel - Relaxed (PSS – upper row) and activated (PDBu – lower row) pig antrum tissue reacted with PKCα (left column, green) and caveolin (middle column, red) and merged with nuclei (right column, PKCα – green; caveolin – red; DAPI – blue). Co-localization of PKCα and caveolin result in yellow/orange fluorescence. Scale bar = 10 µm.

**Figure 7:**
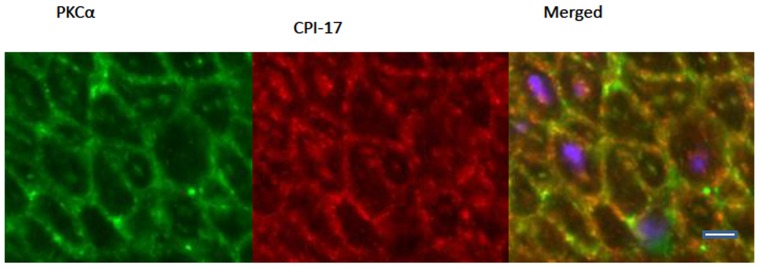
Pig antrum activated with PDBu and reacted with PKCα (left column, green) and CPI-17 (middle column, red) and merged with nuclei (right column, PKCα – green; CPI-17– red; DAPI – blue). Co-localization of PKCα and CPI-17 result in yellow/orange fluorescence. Scale bar = 10 µm.

The primarily peripheral distribution of PKCα (under both relaxed and stimulated conditions) and CPI-17 (following 30′ stimulation with CCh or PDBu) is not uniform at the cell periphery, but punctate in distribution (Fig. 6). There is also a punctate distribution of the anatomically and functionally distinct adherens junctions and caveoli at the SMC plasma membrane [26]. In order to determine if the distribution of the PKCα and CPI-17 at the membrane corresponds with the punctate pattern of adherens junctions, we did double labeling with two adherens junction associated proteins, vinculin and talin. The alternate pattern of distribution of the red and green fluorophores at the membranes show that the PKCα and CPI-17 do not co-localize with either vinculin or talin (Fig. 6), suggesting that the PKCα and CPI-17 do not associate with the adherens junction complex under the conditions we examined. Because caveoli alternate with the adherens junctions at the peripheral membrane [26], PKCα and CPI-17 may co-localize with the caveoli. To determine if the punctate distribution of the PKCα and CPI-17 at the membrane corresponds with the punctate pattern of the caveoli, we also did double labeling of PKCα with caveolin. The distribution of PKCα and caveolin co-localize at the membrane in both relaxed and activated conditions as can been observed by the appearance of the yellow/orange fluorescence rather than distinct red and green fluorescence (Fig. 6). A two-tailed F-test for equality of two standard deviations (SDs) determined a significantly lower SD in the ratio of caveolin to PKCα when compared to the SD of the ratio of vinculin to PKCα (n = 20 cells; p<0.05). In addition, double labeling of PKCα with CPI-17 following activation of the tissue also shows significant co-localization (n = 20 cells; p<0.05) of these two proteins near the plasma membrane (fig. 7). These data show that PKCα is primarily localized near the caveloi at the SMC plasma membrane at all times, and that following tissue activation, a significant portion of the cytosolic CPI-17 translocates to the caveoli at the plasma where it co-localizes with PKCα.

## Discussion

Smooth muscle tissues are routinely characterized as being either “tonic” (generating a slow maintained isometric contraction) or “phasic” (generating a fast transient isometric contraction). Force generation and/or tissue shortening in SM are believed to require elevated myosin light chain phosphorylation, ATP consumption and cross bridge cycling. A possible mechanism responsible for these two types of contractions is differential Ca^2+^ sensitization. Ca^2+^ sensitization in smooth muscle is believed in large part to be due to the inhibition of MLCP concurrent with or subsequent to the activation of MLCK during tissue activation [15], [35]. One of the major pathways proposed to be involved in Ca^2+^ sensitization is via G-protein coupled receptors (GPCR)/phospholipase C (PLC)/diacylglycerol (DAG)/PKC/CPI-17 to inhibit MLCP. GPCR’s, PLC, and DAG are all located at/in the plasma membrane. The location of the MLCP is less certain, but at some point in time following tissue activation MLCP needs to be associated with MLC_20_ on the myosin molecule of the thick filaments in order to dephosphorylate this protein. Because thick filaments have been reported to be distributed throughout the SMC cytoplasm [36], [37], [38], [39], the MLCP will also need to be distributed throughout the cytoplasm at some point during relaxation [40]. If MLCP is located within the cytosol and near MLC_20_ during contraction, and PKCα and CPI-17 play a role in inhibiting MLCP during contraction, then at least CPI-17 would be expected to be located within the cytosol at some time during contraction. The results of this study show that PKCα is preferentially localized near the plasma membrane during relaxed and activated conditions and that CPI-17 is also preferentially localized near the plasma membrane during activated conditions. Thus, these data suggest that differences in the PKCα- CPI-17 Ca^2+^ sensitization pathway cannot explain tonic and phasic SM contractions in stomach fundus and antrum and further work is required to determine how the spatial-temporal distribution of CPI-17 with tissue activation can regulate MLCP to cause Ca^2+^ sensitization of SM contraction in intact SM tissues.

While PDBu causes a slow contraction in the fundus which is ∼40% of the KPSS induced peak force, little contraction is generated in the antrum with PDBu stimulation. Since fundus and antrum represent two basic types of smooth muscle, tonic and phasic respectively, it seems logical to ascribe the difference in force generation to distinct properties of phasic and tonic muscle. Woodsome et al [41] reported that the expression level of CPI-17 in tonic vascular SM is higher than that in phasic vascular SM. This could explain the different ability of tonic muscles to maintain force (a high concentration of CPI-17 inhibits MLCP allowing MLC_20_ phosphorylation to stay high and force to be maintained in tonic SM). This study examined PKCα and CPI-17 protein expression and distribution in the visceral swine stomach. To our surprise, no significant difference in the protein expression level of PKCα or CPI-17 was detected between the fundus and antrum (Fig. 2). These results indicate that the difference between tonic and phasic smooth muscle in responding to varied stimulation cannot be explained solely by differences in proteins expression of PKCα or CPI-17 in swine stomach SM tissues.

Studies on the spatial-temporal distribution of PKC remain controversial. Several studies suggest that PKC works as a moving messenger to relay the cell activation signal from the membrane to the cytosol. PKCα, a member of a large serine/threonine family of kinases, was first identified in 1977 by Nishizuka’s group [42], [43]. Castagna et al [34] who were the first to report that tumor-promoting phorbol esters could also cause direct activation of the Ca^2+^ activated, phospholipid-dependent PKC marking its significance and relevance in affecting cellular function. The first report of PKC’s cellular localization was in EL4 mouse thymoma cells where PKC activity was reported to be high in the cytosolic fraction but was reduced following phorbol ester stimulation [44]. Subsequent biochemical studies in smooth muscle reported that PKCα translocates to the membrane at different times and for different lengths of time with different agonists [45], [46], [47].

Immunofluorescence microscopy, which allows direct observation of potential PKC movement within cells, shows that PKCα undergoes a process of relocation from the cytosol to the plasma membrane after agonist stimulation in isolated single smooth muscle cells [48], [49], [50], [51], [52]. In contrast to these studies, there is also evidence for movement of PKC in the opposite direction. Fay’s group [53] reported that in isolated toad stomach single cells, a pool of activated PKC was identified adjacent to the plasma membrane. Upon stimulation with CCh, activated PKC (no specific isoform mentioned) was released from the plasma membrane associated pool and redistributed throughout the cytosol. Further study showed that PKC became associated with contractile filaments after the treatment [53].

To eliminate potential changes that can occur in isolated/cultured cells compared with multicellular preparations (cell isolation procedures disrupt cell-cell associations and alter/remove the extracellular matrix allowing SM cytoskeletal changes) [54], [55], intact tissues were used in this study to determine the PKCα distribution in smooth muscle cells during relaxation and contraction. Surprisingly, the PKCα distribution was not “uniform” throughout the cell in relaxed conditions nor was it changed upon stimulation in intact tissues. PKCα was primarily distributed adjacent to the plasma membrane, before and after PDBu or CCh treatment (Fig. 3). Since PKCα is a fairly large protein, it may not be efficient to use as a moving second messenger within the cell. CPI-17 however is a small soluble protein down-stream from PKCα that should be able to freely diffuse throughout the SMC until PKCα is activated and binds the CPI-17. Binding of CPI-17 by PKCα at the membrane would change the concentration of the free CPI-17 in the subplasmalemmal region causing more CPI-17 to diffuse to the cell periphery where it also could be bound by activated PKCα. In this way no active means of transporting CPI-17 to the periphery would be necessary, as simple mass diffusion due to the altered free/bound concentration of CPI-17 could account for this “translocation”. Our data are consistent with this, showing a diffuse distribution of CPI-17 in SMCs under relaxed conditions and a preferential peripheral distribution following activation of the tissue (Fig. 4 & 5). This translocation of CPI-17 occurs in both the tonic and phasic stomach fundus and antrum and thus cannot account for the difference in their contraction patterns. In addition, the 3–30 minutes that it takes for the CPI-17 to show a significant translocation to the membrane appears to be too slow to account for the early tonic force observed in tonic muscles. This does not however exclude the possibility that this pathway is involved in the longer maintenance of tonic force.

It is not clear what keeps the PKCα preferentially at the plasma membrane. DAG is membrane bound and can function as a link to PKCα at the cell membrane while it is activating PKCα. However the accumulation of PKCα at cell periphery under resting conditions observed in this study raises a question about the primarily peripheral distribution of PKCα when [DAG] is presumed to be low. Since there is no evidence to support PKCα as a membrane embedded protein, it is necessary to speculate other membrane associated proteins binding to PKCα under relaxed conditions to prevent it from randomly diffusing throughout the cell. Several proteins have been proposed to have the ability to hold PKCα adjacent to the cell membrane. It was reported that in cultured cells, PKCα could also be detected in focal contact structures and might bind to talin or vinculin [56]. In our study, double labeling of PKCα with talin or vinculin in intact swine stomach tissues shows PKCα preferentially near the plasma membrane but in an alternate punctate pattern with either vinculin or talin (Fig. 6), suggesting that PKCα is not associated with the adherens junction.

Several other studies suggest PKCα could be a resident protein in a membrane structure called caveolae [57], [58], [59], [60]. Studies have identified multiple caveolae-associated proteins as having the potential to bind to PKCα. For instance, a 68 kD PKCα binding protein termed serum deprivation response (sdr) was localized in caveolae [59]. It was also reported that PKCα has a particular amino acid sequence (522WAYGVLLY528) in the catalytic domain which has the potential of interacting with the scaffolding domain of Caveolin-1, a resident protein in caveolae [61]. This observation is also supported by *in vitro* assays suggesting caveolin-1 has an inhibitory effect on PKCα. Sucrose gradient centrifugation showed Caveolin-1 and PKCα are accumulated in the same fraction, which might suggest a bond between the two proteins [62].

Several membrane receptors termed receptors for inactive/active C-kinase isozymes (RICKs and RACKs) were also proposed to be able to interact with inactivated or activated PKC [21], [22], [63]. Their function is expected to maintain PKC in distinct locations on or near the cell membrane. One of these scaffolding proteins, AKAP150, has been reported to be able to target PKC to unique plasma membrane domains where L-type Ca^2+^ channels are located. The contact between PKC and Ca^2+^ channels was suggested to be required for generating constitutive Ca^2+^ influx [64]. This is consistent with the observation that a population of L-type Ca^2+^ channels is localized to caveolae in ventricular myocytes, since PKC was also predicted to reside in the same location [65] and has been reported for muscarinic smooth muscle [66]. It seems logical to propose that PKCα is anchored by caveolin- or caveolae-associated protein(s) to the cell membrane and activated by DAG there. Our data showing that PKCα co-localizes with caveolin at the caveolae (Fig. 6), and in an alternating punctate pattern with vinculin/talin, is consistent with this idea.

The primarily peripheral distribution of CPI-17 following tissue activation might be explained by its binding to peripherally located, activated, PKCα. Sakai et al. examined the spatial-temporal regulation on CPI-17 in bronchial smooth muscle of rats under acetylcholine (ACh) stimulation [67]. Their immunoblotting experiments revealed a time-dependent shift of CPI-17 from the cytosolic fraction to the membrane fraction upon stimulation and phosphorylated CPI-17 was only found in the membrane fraction, suggesting that CPI-17 is activated at the plasma membrane [67].

Our study also revealed a punctate distribution of CPI-17 at the membrane after stimulation (Fig. 7). The exact association of CPI-17 at the membrane is not known at this time, but CPI-17 does co-localize with PKCα at the caveolae. This seems logical as PKC and CPI-17 need to be associated with each other at some point if PKCα is going to phosphorylate CPI-17. And while CPI-17 is observed to translocate from the cytosol to the membrane upon stimulation (Fig. 4 & 5), there is still a significant amount of CPI-17 remaining in the cytosol following tissue activation (Fig. 4 & 5 and [68]). Kolosova et al. [68] reported that the distribution of CPI-17 was parallel with actin in human pulmonary artery endothelial cell suggesting a co-localization of CPI-17 and actin. Thus it is possible that when CPI-17 is in the cytosol, it associates with actin filaments, though we saw no evidence for this in our tissues. Moreover, a certain amount of CPI-17 has been reported to be located adjacent to the nucleus [69]. This may be explainable by reports that PKCα can localize to the perinuclear region in vascular smooth muscle cells [70].

Our data show that the translocation of CPI-17 to the membrane is a very slow process requiring greater than 3 min for a significant shift (Fig. 4) and that it remains preferentially localized near the plasma membrane following 30 min of stimulation with either CCh or PDBu. Sakai et al [67] reported that it takes two minutes following ACh stimulation for CPI-17 to translocate to the membrane fraction in rat bronchial SM and that it remains there as long as the tissue is stimulated (>20 minutes). They also reported the presence of phosphorylated CPI-17 follows a slightly slower time frame, becoming significant in the membrane fraction at ten minutes. Phosphorylated CPI-17 was not observed in the cytosolic fraction, and only decreased in concentration following relaxation of the tissue. Thus our data (fig. 4) and that from [67] suggests that CPI-17 (p-CPI-17) does not return to the cytosol (cytosolic fraction for Sakai) while the tissue is activated.

The failure of CPI-17 (p-CPI-17, [67]) to return to the cytosol during activation of SM tissue leaves a gap in the PKC/CPI-17/MLCP pathway for Ca^2+^ sensitization via inactivation of MLCP. While CPI-17 (p-CPI-17, ref [67]) is primarily localized near the plasma membrane following activation of smooth muscle tissues [67] the MLC_20_ is distributed throughout the cell as part of the thick filament myosin protein. MLCP needs to be associated with myosin if it is going to dephosphorylate MLC_20,_ and it needs to be associated with CPI-17 if it is going to be inhibited from dephosphorylating MLC_20_. There are a number of possible explanations to address this. The first would be that there is another currently unidentified protein(s) in this pathway that is activated by CPI-17 and is responsible for inhibiting MLCP. This seems unlikely based on publications showing a direct inhibitory effect of CPI-17 on MLCP [71]. A second possibility would be that CPI-17 is not involved in Ca^2+^sensitization in intact visceral SM tissues, or perhaps not in specific SM tissues (stomach and bronchial). This seems equally unlikely as there are reports of this pathway being relevant in the gut and airway [17], [67]. A third possible explanation would be that MLCP actually translocates to the periphery by mass action following the shift in distribution of CPI-17. It could be inactivated by CPI-17 at the periphery, or merely no longer effective as a MLC_20_ phosphatase because it is no longer associated with MLC_20_ on the thick filaments located in the cytosol [40], [72]. This last explanation seems to warrant further testing.

This leads us to propose the following model for Ca^2+^ sensitization via the PKC/CPI-17/MLCP pathway. In intact visceral (and other?) smooth muscle tissues, PKCα is localized primarily at the plasma membrane by binding to RICKs [21]. With tissue activation, an increase in plasma membrane DAG activates PKCα at the membrane. CPI-17 is bound by the activated membrane bound PKCα, causing a shift in its distribution from diffusely distributed throughout the cytosol to primarily at the plasma membrane. Activated PKCα activates the bound CPI-17 (p-CPI-17) which remains at the membrane until the activating stimulus is removed and the tissue relaxes. How the MLCP is inhibited, and whether it moves within the cell remains unresolved. We hypothesize that the p-CPI-17 binds to MLCP at the plasma membrane. This would have two consequences. The first is that there would be a shift in distribution of MLCP from the cytosol to the plasma membrane (we were unable to test this as no MLCP antibodies could be obtained that worked in the swine tissue). The second consequence would be that by shifting the distribution of MLCP away from myosin (MLC_20_) to the plasma membrane, MLCP would be ineffective in de-phosphorylating MLC_20_. This would explain the Ca^2+^ sensitization by keeping MLC_20_ phosphorylation levels elevated and enhancing tissue force during the extended period of time the tissue is stimulated.

A remaining unresolved issue is the different responses of the tonic fundus and phasic antrum to PDBu stimulation. One possibility is that the different responses to PDBu are caused by differences in basal Ca^2+^ level between tonic and phasic smooth muscle. It was reported that tonic tissues have a higher level of [Ca^2+^]_i_ than phasic tissues under relaxed conditions [73]. It is possible that although PDBu administration could boost Ca^2+^ sensitization in phasic smooth muscle to a similar level as in tonic tissues, the low basal level of [Ca^2+^]_i_ might still not be able to maintain a strong contraction in phasic tissues. The differences of sarcoplasmic reticulum distribution in the two types of tissues may be responsible for differences in the [Ca^2+^]_i_ in basal conditions [74]. A second possibility could be that expression patterns of MLCP are different between tonic and phasic smooth muscle and this difference leads to differences in contraction to PDBu stimulation. This is supported by the observation that phasic smooth muscle contains more MLCP than tonic tissues [41]. The above explanations are not necessarily exclusive to each other and the cause for differences in force generation between tonic and phasic SM may be the combination of the two, and further study is needed to address these issues. In addition, there are numerous other second messenger pathways reported to affect MLCP activity including RhoA/ROCK, ILK, and Zip kinase, [18] and these undoubtedly are also important and need to be tested. While the entire mechanism remains unresolved, this study shows there are no differences in PKCα and CPI-17 protein expression or distribution that can explain the sustained tonic vs. transient phasic contractions observed in intact stomach fundus and antrum smooth muscle.

## Supporting Information

Figure S1 **Representative confocal Z-stack series of CPI-17 distribution in transverse section of the circular layer of pig antrum in relaxed conditions (PSS).** Tissues were immunoreacted for CPI-17 (green). CPI-17 appears diffusely distributed throughout the cell regardless of the level of the Z-stack section in relaxed conditions.
(TIF)

Figure S2 **Representative confocal Z-stack series of CPI-17 distribution in transverse section of the circular layer of pig antrum following 30 minutes stimulation in 1** **µM PBDu. Tissues were immunoreacted for CPI-17 (green).** With PDBu stimulation, CPI-17 appears predominantly located at the periphery near the plasma membrane regardless of the level of the Z-stack section.
(TIF)
