# Supplementary figures and images for: Tonic and Phasic Smooth Muscle Contraction Is Not Regulated by the PKCα - CPI-17 Pathway in Swine Stomach Antrum and Fundus
Source: PLoS One. 2013 Sep 18;8(9):e74608. doi: 10.1371/journal.pone.0074608 (PMC3776813; doi:10.1371/journal.pone.0074608)

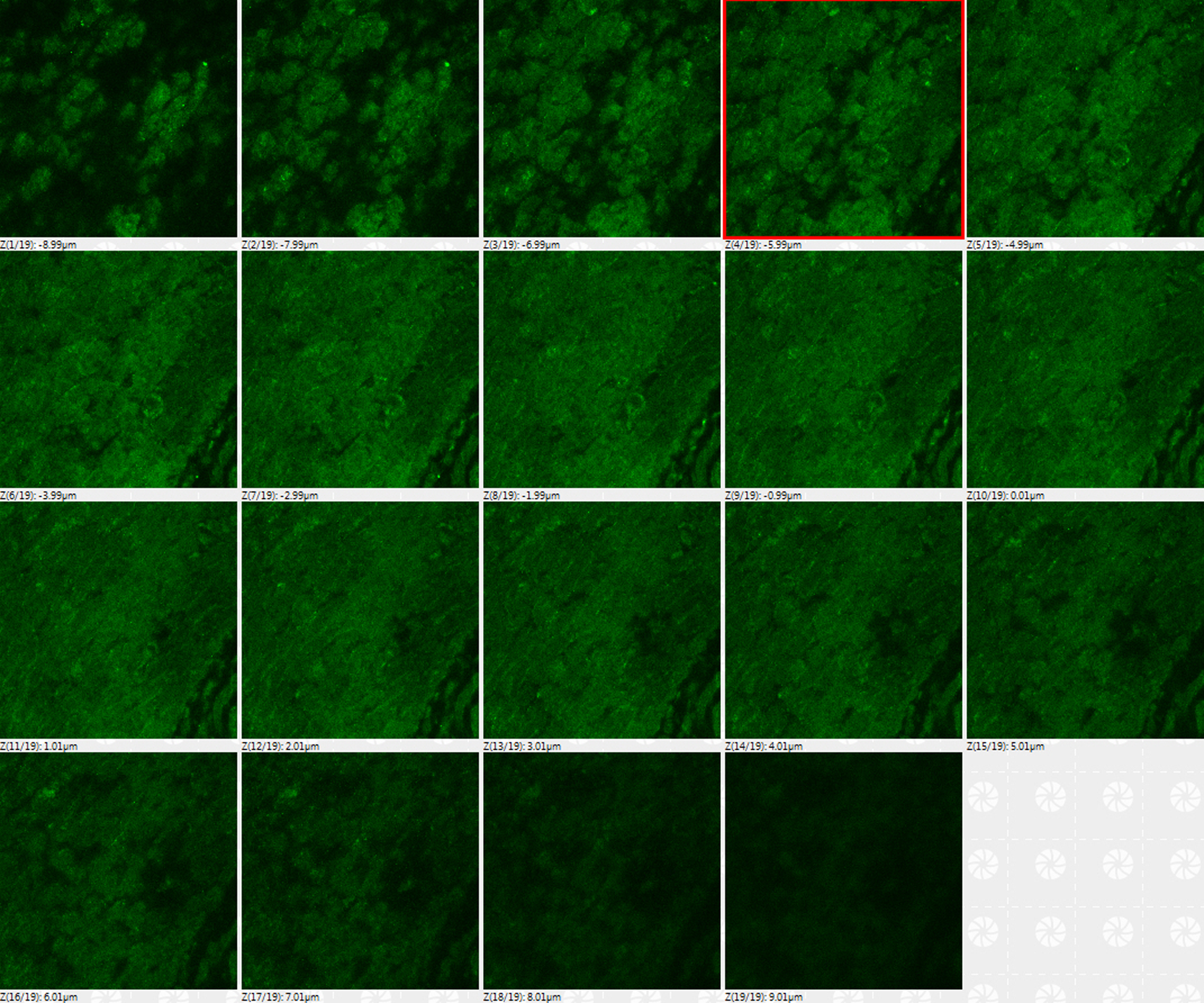

Supplement: Figure S1 — Representative confocal Z-stack series of CPI-17 distribution in transverse section of the circular layer of pig antrum in relaxed conditions (PSS). Tissues were immunoreacted for CPI-17 (green). CPI-17 appears diffusely distributed throughout the cell regardless of the level of the Z-stack section in relaxed conditions. (TIF) [file pone.0074608.s001.tif]

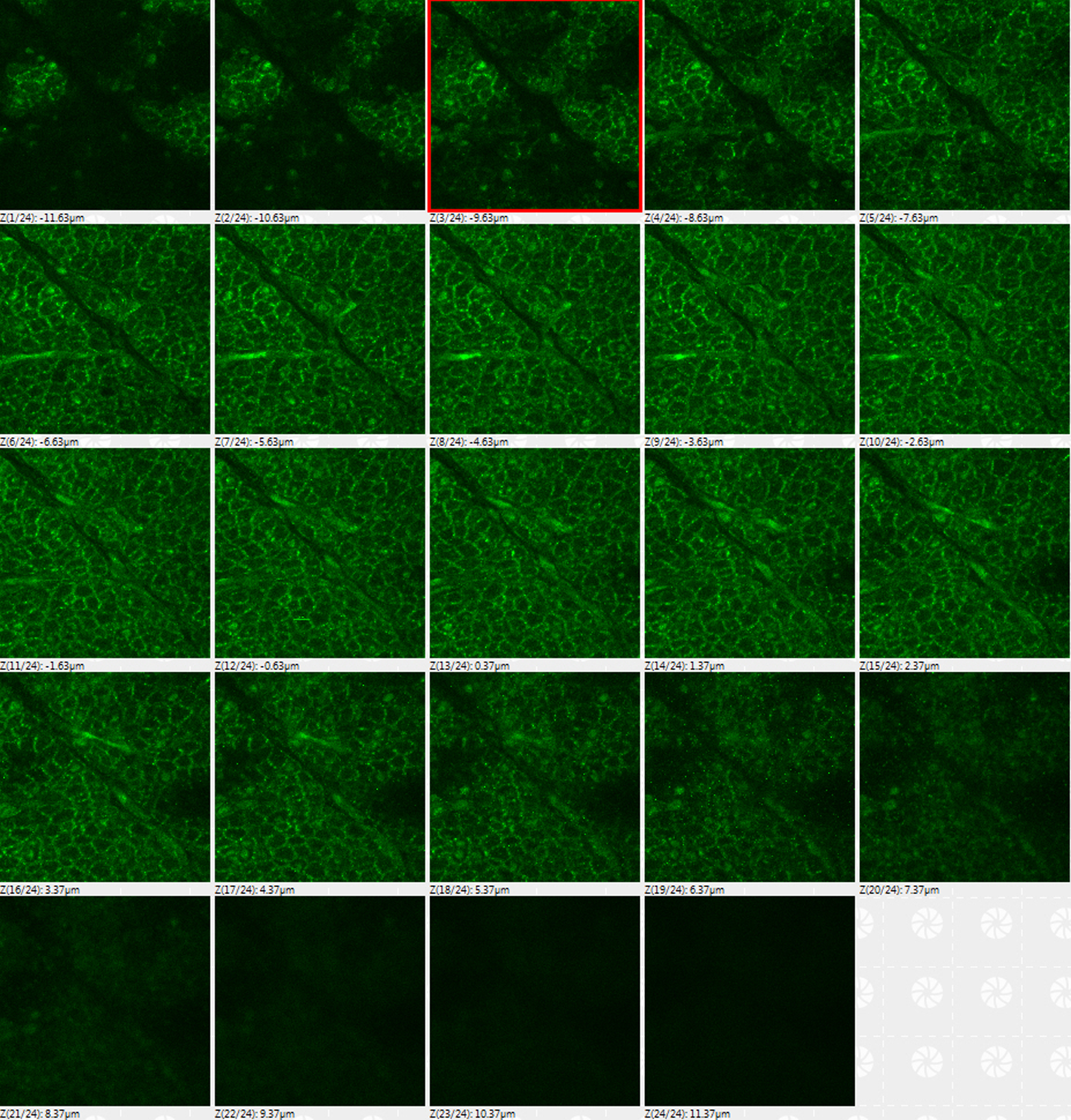

Supplement: Figure S2 — Representative confocal Z-stack series of CPI-17 distribution in transverse section of the circular layer of pig antrum following 30 minutes stimulation in 1 µM PBDu. Tissues were immunoreacted for CPI-17 (green). With PDBu stimulation, CPI-17 appears predominantly located at the periphery near the plasma membrane regardless of the level of the Z-stack section. (TIF) [file pone.0074608.s002.tif]
